# Correlation Between Anterior Chamber Volume and Corneal Biomechanical Properties in Human Eyes

**DOI:** 10.3389/fbioe.2019.00379

**Published:** 2019-12-03

**Authors:** Xinhan Cui, Yujing Yang, Yue Li, Feifei Huang, Yujin Zhao, Huiyu Chen, Jianjiang Xu, Alireza Mashaghi, Jiaxu Hong

**Affiliations:** ^1^Department of Ophthalmology and Visual Science, Eye and ENT Hospital of Fudan University, Shanghai, China; ^2^Leiden Academic Centre for Drug Research, Leiden University, Leiden, Netherlands; ^3^Key Laboratory of Myopia, Ministry of Health (Fudan University), Shanghai, China; ^4^Shanghai Key Laboratory of Visual Impairment and Restoration, Fudan University, Shanghai, China; ^5^Department of Ophthalmology, The Affiliated Hospital of Guizhou Medical University, Guiyang, China

**Keywords:** cornea, biomechanical properties, anterior chamber, primary angle closure, imaging

## Abstract

**Purpose:** To investigate the correlation between anterior chamber volume (ACV) and corneal biomechanical properties in healthy and primary angle closure (PAC) eyes.

**Methods:** A total of 79 eyes from 55 participants were enrolled in this study, including 24 eyes from 17 PAC patients and 55 eyes from 38 normal subjects. Anterior chamber volume was detected via swept-source anterior segment optical coherence tomography (OCT), and the cornea biomechanical data were obtained via corneal visualization Scheimpflug technology (Corvis ST). A student's *t-*test, Chi-square test, Pearson's correlation coefficients, and linear regression were used in the statistical analysis.

**Results:** Anterior chamber volume was significantly associated with a lower deformation altitude (DA) (*p* = 0.033), higher stiffness parameter (SP-A1) (*p* = 0.005), younger age (*p* = 0.001), and higher biomechanical intraocular pressure (bIOP) (*p* = 0.001). PAC patients were suspected to have a much shallower anterior chamber than healthy participants. In the PAC group, the mean ACV (*p* < 0.01), SP-A1 (*p* < 0.01), and bIOP values (*p* = 0.01) were significantly reduced as compared to the normal group, and DA values (*p* = 0.02) and age (*p* = 0.01) were increased as compared to the normal group.

**Conclusions:** Bigger ACV was associated with lower DA values and age, as well as higher SP-A1 and bIOP values. Reduced corneal stiffness was more commonly found in the PAC suspects as compared to their normal counterparts, indicating a protective physiological mechanism for people with shallower anterior chambers that protects against potential elevations of IOP.

## Introduction

The cornea provides a proper anterior refractive surface for the eye and protects the intraocular contents from both infection and structural damage. The biomechanical properties of the cornea are important in maintaining corneal health. The measurement of these parameters helps in diagnosing certain diseases, such as keratoconus, Fuch's dystrophy, and glaucoma (del Buey et al., 2009; Medeiros et al., 2013; Elham et al., 2017). In addition, the therapeutic manipulation of corneal biomechanics has been introduced as a treatment strategy for ectatic corneal diseases (Wollensak et al., 2003) and presbyopia among others (Krueger, 2009). Thus, an understanding of corneal biomechanics is important in ophthalmology, both for diagnostics and therapeutics.

Measurements of human corneal biomechanics are traditionally destructive and performed after tissue excision (Lombardo et al., 2014). *In vivo* corneal biomechanics assessment began in 2005 with the introduction of the ocular response analyzer (ORA; Reichert Ocular Instruments, Depew, NY) (Luce, 2005). The ORA is a bidirectional applanation device that allows the indirect assessment of corneal deformation based on infrared light signals. Another method, called corneal visualization Scheimpflug technology (Corvis ST; Oculus Inc., Wetzlar, Germany), applies an additional high-speed Scheimpflug camera to detect changes in corneal shape (Naderan and Jahanrad, 2017; Chan et al., 2018).

Anatomically, the anterior chamber serves as the rear support for the cornea and may also have some effects on corneal biomechanics. Swept-source anterior segment OCT (Casia SS-1000 OCT; Tomey, Nagoya, Japan) has a high scan speed and distinct axial resolution, providing representative imaging of the cornea, iris, and lens for the measurement of anterior chamber volume (Mak et al., 2013). To date, there have been relatively few reports focused on the biomechanical properties of the cornea and their association with the depth or volume of the anterior chamber. Evidence suggests that anterior chamber depth has a negative correlation with corneal hysteresis (CH) (Chang et al., 2010). In contrast, other studies have demonstrated no such association (Hwang et al., 2013; Nemeth et al., 2017). To resolve this issue, proper sample selection and study design are required.

Therefore, the purpose of this study was to investigate the relationship between anterior chamber volume as obtained via Casia SS-1000 OCT and specific corneal deformation parameters determined by Corvis ST.

## Methods

### Subjects

To examine a wide range of ACV values, both healthy participants and primary angle closure (PAC) patients were chosen as the target in this study. The study was conducted in the Eye and ENT Hospital of Fudan University, Shanghai, China. All the subjects underwent a thorough examination of their visual acuity and intraocular pressure (IOP), as well as slit-lamp ophthalmic examination, fundus examination, optical coherence tomography (OCT), and Corvis-ST test (Oculus, Wetzlar, Germany). The details of the period are listed as following: visual acuity (15 min), intraocular pressure (10 min), slit-lamp ophthalmic examination (20 min), fundus examination (15 min), OCT (25 min), and Corvis-ST test (15 min). Usually, the interval between each examination is more than 10 min. The exclusion criteria were any ocular trauma, anterior segment disease, any intraocular, or refractive surgery. To eliminate the possible influence of refractive errors to anterior chamber, participants with refractive power beyond ±1.5 D were excluded. For the purposes of this study, PAC eyes were defined as eyes in which appositional contact between the peripheral iris and posterior trabecular meshwork (<180 of visible trabecular meshwork upon gonioscopy) without peripheral anterior synechia was detected during gonioscopy or OCT (Foster et al., 2002; Mak et al., 2013). The protocol was in accordance with the ethical standards stated in the 1964 Declaration of Helsinki and approved by our hospital ethics committee. After informed consent was obtained, a total of 79 eyes from 55 participants were enrolled, including 24 eyes from 17 PAC patients and 55 eyes from 38 normal subjects.

### Swept-Source Anterior Segment Optical Coherence Tomography

**Figure 1:**
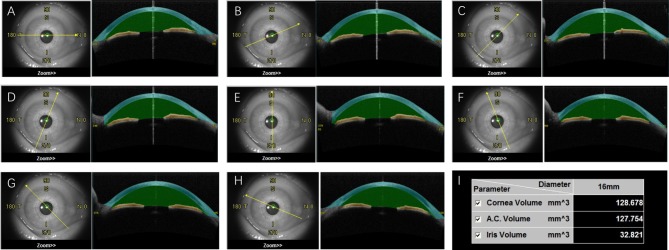
The anterior chamber volume (ACV) automatically calculated by OCT. After the scanning, OCT images were divided into eight sections with different orientations **(A–H)**. The space between cornea (blue) and iris (yellow) was defined as anterior chamber (green). The volume of cornea, anterior chamber and iris were then automatically calculated **(I)**.

Casia SS-1000 OCT (Tomey, Nagoya, Japan) with a swept-source laser wavelength of 1,310 nm was chosen to measure ACV in this study. This method uses a monochromatic, tunable, fast-scanning laser source, and a photodetector to detect wavelength-resolved interference signals (Yasuno et al., 2005; Liu et al., 2011; Mak et al., 2013). The ACV scan protocol contains 128 radial scans, each 16 mm in length and 6 mm in depth. The participants were asked to fixate on an internal fixation target during the scan. With the technician's help, they elevated the upper lid against the upper orbital rim and lowered the lower lid against the lower orbital rim to expose the entire limbus. After a scan duration of about 2.4 s, 360° of the anterior segment were automatically scanned and saved by the program. Each eye was imaged three times. A single masked observer was selected to measure ACV in all of the image series. The ACV value was automatically calculated by the instrument's software (Figure 1).

### Corneal Visualization Scheimpflug Technology

The biomechanical properties of the cornea were measured via corneal visualization Scheimpflug technology, Corvis-ST. The device contains an ultra-high-speed Scheimpflug camera that captures 140 frames within 31 ms with a resolution of 640 × 480 pixels and covers 8.5 mm of the central cornea. While in operation, this device applies a precise air impulse with a Gaussian intensity distribution and a duration of 25 ms to the corneal apex. This air puff causes the cornea to move inwards, creating the first applanation point (applanation 1) and then a slight concavity. When the pressure from the air puff decreases, the cornea gradually returns to its normal convex curvature, passing through a second applanation point (applanation 2) (Salvetat et al., 2015). The Corvis-ST printout provides IOP and CCT measurements, and 13 numerical corneal deformation parameters are listed in Table 1 (Salvetat et al., 2015; Joda et al., 2016).

**Table 1:** Corneal biomechanical parameters of Corvis ST.

**Parameters**	**Definition**
A1L	Cord length of the first-degree applanation
A1V	Corneal speed during the first-degree applanation
A2L	Cord length of the second-degree applanation
A2V	Corneal speed during the second-degree applanation
PD	Distance of the 2 knee's at highest concavity
Radius	Central concave curvature at highest concavity
DA	Maximum amplitude at the apex of highest concavity
CCT	Central corneal thickness
IOP	Intraocular pressure (uncorrected non-contact tonometer)
bIOP	biomechanically-corrected IOP
DARatio	The ratio between the deformation/deflection amplitude at the apex and the average deformation/deflection amplitude measured at 1 or 2 mm from the center
IR	Integrated radius, the inverse concave radius (1/R) between the first and second application events
ARTh	Ambrosio's Relational Thickness to the horizontal profile, the devision between corneal thickness at the thinnest point and the Pachymetric Progression.
SP-A1	Stiffness parameter at first applanation, the resultant pressure (Pr) decided by deflection amplitude at first applanation (A1)
CBI	Corvis Biomechanical Index, a combined biomechanical index based on different dynamic corneal response parameters, to aid the diagnosis of ectasia

### Statistical Analysis

The statistical analysis was performed using SPSS Version 20 (SPSS Inc. Chicago, IL, USA). Descriptive statistical results were written as mean ± standard deviations (SDs). A Pearson's correlation analysis was used to investigate the relationship between ACV and demographic/corneal biomechanical parameters. A multivariate linear regression analyses were performed using any significantly changed parameters as independent variables. A student's *t-*test and Chi-square test were used to compare the differences between PAC patients and healthy participants. The correlation coefficients were recorded, and a *P*-value below 0.05 was considered statistically significant.

## Results

Our study enrolled 79 eyes from 55 participants, including 24 eyes from 17 PAC patients and 55 eyes from 38 normal subjects. The demographics, ACV values, and corneal biomechanical parameters of the participants (mean ± SD and range) were shown in Table 2. The average ACV value was 140.83 ± 38.11 mm^3^, and these values ranged from 79.58 to 256.93 mm^3^.

**Table 2:** Demographics, ACV and corneal biomechanical parameters of the participants, and comparisons between the PAC patients and the normal group.

	**Total (*n* = 79)**	**PAC patients (*n* = 24)**	**Normal (*n* = 55)**	***P*-value**
	**Mean ± SD (Range)**	**Mean ± SD**	**Mean ± SD**	
Age (y)	52.34 ± 14.34 (22–86)	57.96 ± 10.92	49.89 ± 15.04	0.01^**^
Sex (M/F)	26/53	7 / 17	19 / 36	0.64
Height (cm)	162.15 ± 8.38 (145–178)	160.78 ± 6.27	162.72 ± 9.11	0.28
Weight (kg)	62.63 ± 10.28 (45–92)	61.26 ± 8.46	63.21 ± 10.97	0.45
ACV (mm^3^)	140.83 ± 38.11 (79.58–256.93)	106.00 ± 26.75	156.02 ± 31.92	<0.01^**^
A1L (mm)	2.25 ± 0.32 (1.50–2.85)	2.15 ± 0.37	2.30 ± 0.29	0.10^**^
A1V (ms)	0.14 ± 0.02 (0.10–0.18)	0.15 ± 0.01	0.14 ± 0.02	<0.01^**^
A2L (mm)	1.93 ± 0.37 (1.19–3.23)	1.88 ± 0.28	1.95 ± 0.40	0.44
A2V (ms)	−0.28 ± 0.03 (−0.34 to −0.14)	−0.28 ± 0.02	−0.27 ± 0.03	0.15
Peak Distance (mm)	4.90 ± 0.21 (4.31–5.33)	4.93 ± 0.19	4.89 ± 0.22	0.46
Radius (mm)	7.18 ± 0.77 (5.56–9.25)	7.02 ± 0.59	7.25 ± 0.82	0.21
DA (mm)	1.05 ± 0.10 (0.77–1.29)	1.09 ± 0.09	1.03 ± 0.09	<0.01^**^
CCT (μm)	548 ± 33.86 (463–636)	533.33 ± 34.80	554.16 ± 31.72	0.01^*^
IOP (mmHg)	15.43 ± 3.02 (9.50–21.50)	14.91 ± 2.10	16.09 ± 2.55	<0.01^**^
bIOP (mmHg)	14.30 ± 2.48 (9.60–20.50)	14.15 ± 1.87	14.79 ± 2.56	<0.01^**^
DARatio	4.33 ± 0.48 (3.20–5.60)	4.55 ± 0.38	4.23 ± 0.49	<0.01^**^
IR (mm)	8.28 ± 1.05 (6.10–10.50)	8.65 ± 0.89	8.11 ± 1.08	0.04^**^
ARTh (μm)	430.12 ± 91.06 (195.30–714.50)	419.17 ± 91.45	434.70 ± 91.35	0.50
SP-A1	126.04 ± 19.42 (86.40–175.20)	114.61 ± 18.29	131.03 ± 17.86	<0.01^**^
CBI	0.13 ± 0.26 (0.00–1.00)	0.24 ± 0.33	0.08 ± 0.21	0.04^**^

*PAC, primary angle closure; SD, standard deviation; ACV, anterior chamber volume. Other abbreviations of corneal biochemical parameters were listed in Table 1*.

** *P < 0.05 considered statistically significant*.

### Association Between ACV and Corneal Biomechanical Parameters

In the Pearson's correlation, only age (*r* = −0.506, *p* < 0.01) (and not most of the demographic factors, such as sex, height, and weight) was negatively correlated with ACV (Table 3). However, several characteristics of the cornea were associated with ACV. A larger ACV correlated with slower A1V (*r* = −0.363, *p* < 0.01), faster A2V (*r* = 0.278, *p* < 0.01); reduced DA (*r* = −0.406, *p* < 0.01), DARatio (*r* = −0.375, *p* < 0.01), IR (*r* = −0.371, *p* < 0.01), and CBI (*r* = −0.255, *p* = 0.02); and higher IOP (*r* = 0.510, *p* < 0.01), bIOP (*r* = 0.600, *p* < 0.01), and SP-A1 (*r* = 0.388, *p* < 0.01) (Table 3).

**Table 3:** Correlation of ACV measured by Casia SS OCT and demographics, corneal biomechanical parameters.

**Variables**	**Pearson's correlation coefficient**	***P*-value**
Age (y)	−0.506	<0.01^**^
Sex (M/F)	−0.103	0.37
High (cm)	0.211	0.06
Weight (kg)	0.130	0.26
A1L (mm)	0.134	0.24
A1V (ms)	−0.363	<0.01^**^
A2L (mm)	0.118	0.30
A2V (ms)	0.278	<0.01^**^
Peak Distance (mm)	−0.158	0.16
Radius (mm)	0.069	0.54
DA (mm)	−0.406	<0.01^**^
CCT (μm)	0.130	0.25
IOP (mmHg)	0.510	<0.01^**^
bIOP (mmHg)	0.600	<0.01^**^
DARatio	−0.375	<0.01^**^
IR (mm)	−0.371	<0.01^**^
ARTh (μm)	0.137	0.233
SP-A1	0.388	<0.01^**^
CBI	−0.255	0.02^**^

** *P < 0.05 considered statistically significant*.

Next, all the parameters statistically correlated with ACV were thrown into a linear regression model using backward selection. For backward selection, we started with all variables in the model, then iteratively removed the variable with largest *p*-value, until all remaining variables had a significant *p*-value (*p* < 0.05). The results showed that ACV was negatively associated with age (coefficient = −0.334, *p* < 0.01) and DA (coefficient = −0.337, *p* = 0.03), but positively associated with bIOP (coefficient = 1.020, *p* < 0.01) and SP-A1 (coefficient = 0.448, *p* < 0.01; Table 4).

**Table 4:** Factors associated with ACV based on multivariable linear regression model using backward selection.

**Variables**	**Coefficient**	***P*-value**
Age (y)	−0.334	<0.01
DA (mm)	−0.337	0.03
bIOP (mmHg)	1.020	<0.01
SP-A1	0.448	<0.01

*The adjusted R^2^ value of this multivariable linear model was 0.482. P < 0.05 considered statistically significant*.

### Comparison Between PAC Patients and the Normal Group

**Figure 2:**
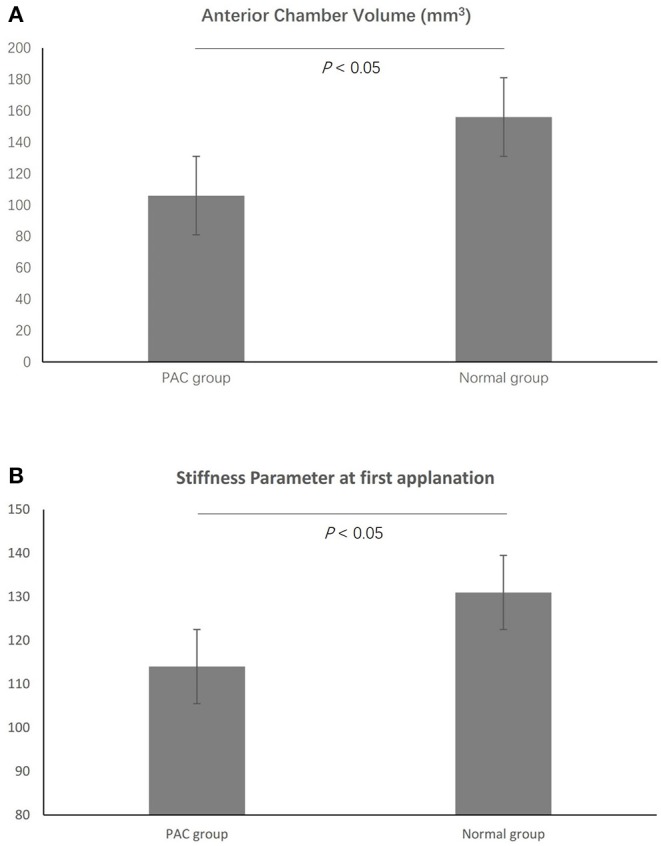
The mean anterior chamber volume (ACV) in primary angle closure (PAC) patients was significantly shallower than in normal participants **(A)**. In addition, the SP-A1 was significantly reduced in the PAC patients as compared to the normal participants **(B)**.

The enrolled PAC patients were slightly older than the participants in the normal group (57.96 ± 10.92 and 49.89 ± 15.04 y, respectively, *p* = 0.01). There were no significant differences in terms of other demographics, such as sex, height, and weight, between the two groups. The mean ACV in PAC patients was 106.00 ± 26.75 mm^3^, significantly shallower than in normal participants (156.02 ± 31.92 mm^3^, *p* < 0.01, Table 2, Figure 2). Besides, it is worth mentioning that the SP-A1 was significantly reduced in the PAC patients as compared to the normal participants (114.61 ± 18.29 and 131.03 ± 17.86, respectively, *p* < 0.01, Figure 2), which was in accordance with the previous linear regression results (Table 2). The other differences between the two groups are listed in Table 2.

## Discussion

This study reported the mean ACV values of PAC patients and normal subjects in a Chinese population, along with their correlated factors. To our knowledge, few such studies have been performed. Measuring these factors with swept-source anterior segment OCT and corneal visualization Scheimpflug technology, we showed that the mean ACV in PAC patients was 106.00 ± 26.75 mm^3^, while in normal participants, this increased to 156.02 ± 31.92 mm^3^. ACV was negatively correlated with age and DA but positively correlated with bIOP and SP-A1. In the group of PAC patients, SP-A1 was significantly reduced.

The correlation between ACV and corneal biomechanics has been discussed in few prior studies. Some showed that anterior chamber depth was negatively correlated with corneal hysteresis, but no significant correlation with corneal resistance factor was observed (Chang et al., 2010). Another study implied that corneal hysteresis was positively associated with the corneal volume, though the association between CH and anterior chamber volume was not significant (Hwang et al., 2013). Regarding corneal biomechanical factors, most of these studies chose to measure corneal hysteresis and corneal resistance factor via ORA (Krueger, 2009; Sedaghat et al., 2017). Similarly, Corvis ST offers parameters about ocular biomechanics as well as ORA and also provides a better understanding of dynamic corneal response (Vinciguerra et al., 2016). To our knowledge, only one paper has calculated the association between ACV and Corvis ST parameters, and this paper reported a negative result (Nemeth et al., 2017). The reason for these conflicting results remains unknown. One possible reason was that the subjects of the studies were different. Nemeth et al. enrolled 43 eyes from Hungarian people, while we collected 79 Chinese eyes. Another possible reason may be due to the different inclusion criteria. To enlarge the range the ACV values, PAC patients were enrolled in this study. Thus, in this study, ACV values ranged from 79.58 to 256.93 mm^3^. Finally, new Corvis ST parameters were discussed in this study which was absent in previous reports. Specifically, several updated parameters, such as DARatio, IR, ARTh, and SP-A1, were discussed, providing a more comprehensive examination of corneal biomechanics.

The average ACV in this study was 106.00 mm^3^ in PAC patients and 156.02 mm^3^ in normal participants. These numbers are in accordance with those found in other studies. Mark et al. investigated Hong Kong citizens, and ACV was found to be 87.6 mm^3^ in PAC patients and 133.0 mm^3^ in normal eyes (Mak et al., 2013). Lam et al. found ACV be 161.03 mm^3^ in 50 healthy middle-aged Chinese people (Sedaghat et al., 2017). In our study, results showed that ACV was negatively correlated with age. Decreases in ACV with age have been reported previously (Lam and Tse, 2013; Hashemi et al., 2016). The reason for this may be thickened crystalline lenses and relaxed accommodation in older people (Lam and Tse, 2013). Except for age, there were multiple corneal biomechanical parameters associated with ACV, as the findings of Pearson's correlation analyses revealed. However, these parameters may have correlations between one and another. To exclude the factors that may be confounding, we built a multivariable linear model to pick up the relevant factors, which may contribute to ACV variations, to strengthen the predicting power. Results showed that ACV was negatively correlated with DA while positively correlated with bIOP and SP-A1. To our knowledge, associations between DA, bIOP, SP-A1, and ACV have been rarely discussed before. Of these factors, SP-A1 shows a consistent and significant association with ACV, as confirmed by the fact that corneal stiffness is reduced in shallow anterior chambers. The novel stiffness parameter SP-A1 is defined as the adjusted pressure at A1 (adjusted AP1) minus a biomechanically corrected IOP value (bIOP) and then divided by A1 deflection amplitude (Vinciguerra et al., 2016). Liu et al. showed that in the stiffened eye, IOP elevations were significantly higher, even at small volume changes, which means that stiffer corneas may be associated with rapid and higher-magnitude IOP fluctuations (Liu and He, 2009).

We note that our study has several limitations. First, the sample selection was biased in some respects. Female subjects were more common than male participants, although the gender imbalance between PAC patients and normal subjects was not significant. Second, the subjects enrolled in the PAC group were older than their normal counterparts. A previous report showed that there was a decrease in corneal stiffness with an increase in age (Sharifipour et al., 2016), which may partly explain why corneal stiffness decreases in our PAC patients. Another limitation of the study is the combined use of the fellow eyes of some participants. Further investigations should be conducted to confirm our results and evaluate SP-A1 in PACG or primary open angle glaucoma (POAG) patients.

In conclusion, our study showed that smaller ACV was associated with decreased DA and age and increased SP-A1 and bIOP in the PAC patients and the healthy subjects. Reduced corneal stiffness was found in the PAC patients as compared to their normal counterparts, indicating a protective physiological mechanism against potential IOP elevations in people with shallower anterior chambers.

## Data Availability Statement

The datasets supporting the conclusions of this article are available from the corresponding author on reasonable request.

## Ethics Statement

The protocol was in accordance with the ethical standards stated in the 1964 Declaration of Helsinki and approved by our hospital ethics committee. After informed consent was obtained, a total of 79 eyes from 55 participants were enrolled, including 24 eyes from 17 PAC patients and 55 eyes from 38 normal subjects.

## Author Contributions

JH, JX, and XC designed the experiment. XC, YY, YL, FH, YZ, HC conducted the experiment. After analyzing the data, XC wrote the initial draft. All authors discussed the analyzed data and the interpretations. JH, JX, and AM revised the manuscript and made the final version.

### Conflict of Interest

The authors declare that the research was conducted in the absence of any commercial or financial relationships that could be construed as a potential conflict of interest.
